# Unilateral Congenital Lacrimal Fistula with Renal Agenesis and Pelvic Kidney: A Case Report and Review of the Literature

**DOI:** 10.1155/2015/368950

**Published:** 2015-05-18

**Authors:** A. Altun, S. A. Kurna, T. Sengor, G. Altun, A. Oflaz, H. S. Sonmez

**Affiliations:** ^1^Fatih Sultan Mehmet Education and Research Hospital, Clinic of Ophthalmology, 34752 Istanbul, Turkey; ^2^Department of Ophthalmology, Bilim University, 34710 Istanbul, Turkey; ^3^Umraniye Education and Research Hospital, Clinic of Pediatrics, 34766 Istanbul, Turkey

## Abstract

A 12-year-old boy presented to the clinic of ophthalmology because of watering and discharge from his left lower eyelid. The inspection examination revealed an orifice that was associated with congenital lacrimal fistula (CLF). He underwent a complete ophthalmologic and systemic evaluation to explore possible associated findings. Systemic evaluation revealed multiple renal anomalies: right renal agenesis and left ectopic pelvic kidney. This case is unique because this is the first reported case of CLF accompanied with ectopic pelvic kidney in the literature.

## 1. Introduction

Rasor described the first reported case of congenital lacrimal fistula (CLF) in 1675 [1]. CLF is a rare abnormality of the nasolacrimal excretory system that is approximately encountered in every 1/2000 birth [2]. CLF might be genetic with autosomal dominant inheritance [2–4], but an autosomal-recessive inheritance pattern has also been reported in the literature [5, 6]. There does not appear to be any race or sex predilection. In this report we would like to present a case of unilateral CLF accompanied with multiple renal anomalies and its surgical management.

## 2. Case Report

**Figure 1:**
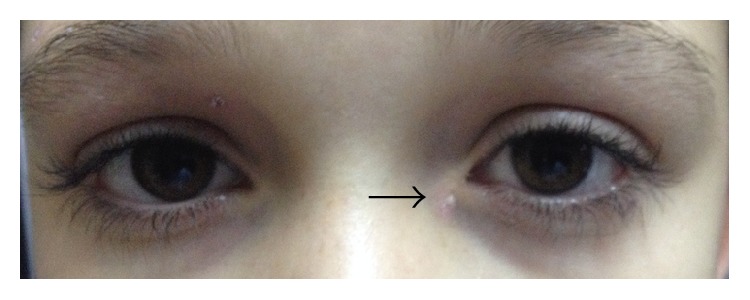
External orifice of congenital lacrimal fistula located inferomedial to medial canthus of the left eye.

A 12-year-old boy presented to the clinic of ophthalmology because of watering and discharge from an orifice close to his left lower eyelid that were increasing on windy days. A lacrimal fistula was observed inferomedial to the medial canthus of his left eye. The opening was spheroidal and approximately 1 mm in diameter. There was a small amount of sebaceous material in the pit, and there was no evidence of excoriation, inflammation, or swelling (Figure 1). We syringed Serum Physiologic through the lower punctum to explore nasolacrimal duct competence. The fluid was passing to the nasopharynx easily and there was no regurgitating through the orifice of fistula. We verified that the fistula was connected to the lacrimal drainage system by probing the fistula. The CLF was connected to the lower portion of lacrimal sac, and there was no combined nasolacrimal duct obstruction or stenosis.

**Figure 2:**
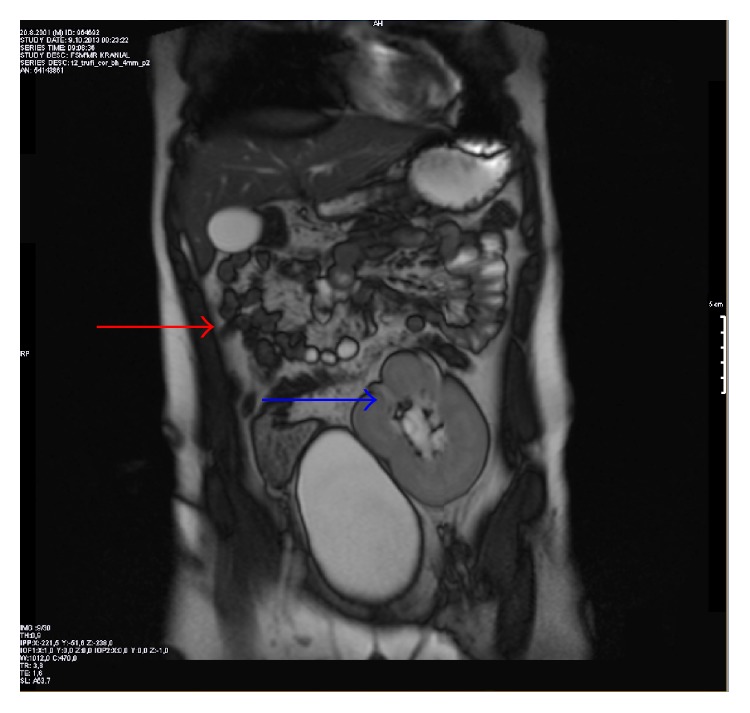
Magnetic resonance imaging of abdomen on the coronal plane. There were right renal agenesis (red arrow) and left ectopic pelvic kidney over the urinary bladder (blue arrow).

**Figure 3:**
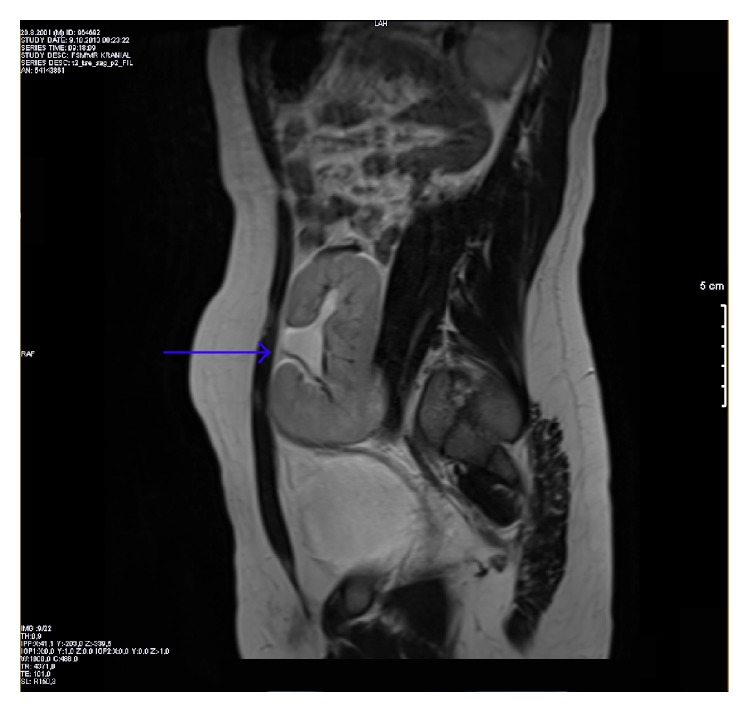
Magnetic resonance imaging of abdomen on the sagittal plane. The left kidney that had one ureter was located over the urinary bladder (blue arrow).

**Figure 4:**
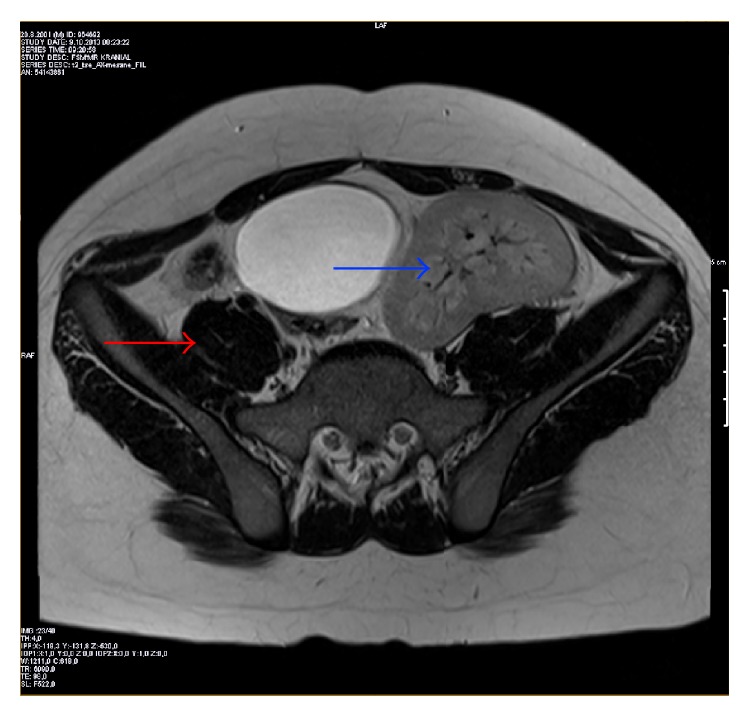
Magnetic resonance imaging of abdomen on the axial plane. There were right renal agenesis (red arrow) and left ectopic pelvic kidney in the neighborhood of urinary bladder (blue arrow).

According to the history obtained from his mother, he had CLF when he was born full term after uncomplicated pregnancy. His family history was negative for any congenital ocular or renal anomalies. Ophthalmic examination revealed uncorrected visual acuity of 20/20 for each eye. The pupils were equal and there was no evidence of a relative afferent pupillary defect. Slit-lamp biomicroscopy revealed normal anterior segments in both eyes. Funduscopic examination, intraocular pressure, and the rest of the ocular findings were within normal limits bilaterally. He underwent systemic evaluation to explore possible associated findings. Systemic evaluation by magnetic resonance imaging revealed multiple renal anomalies: right renal agenesis and left ectopic pelvic kidney on the coronal (Figure 2), sagittal (Figure 3), and axial (Figure 4) planes. The rest of the systemic findings were within normal limits.

**Figure 5:**
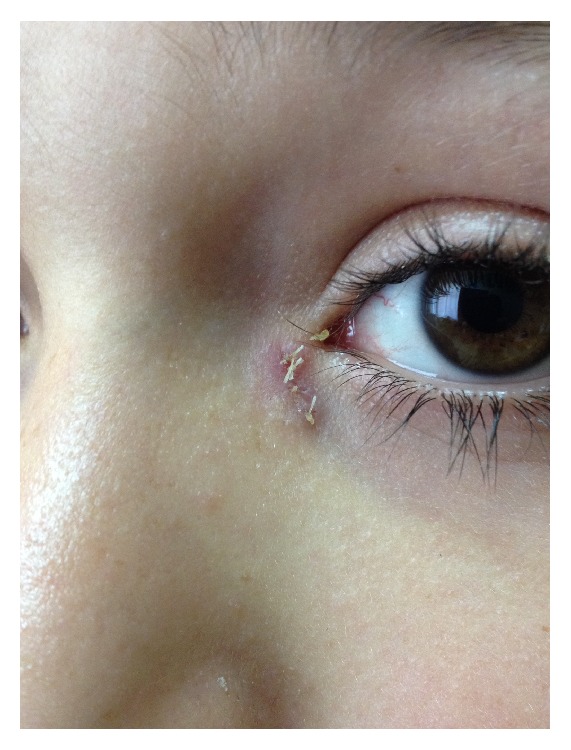
Surrounding tissues were sutured with 6-0 Vicryl after excising the tract of lacrimal fistula.

After performing a fusiform incision around orifice along the skin tension lines, we held the tract with a tooth forceps and dissociated it from the adjacent tissue with Vannas scissors. After dissection we excised the tract partially and sutured surrounding tissue and skin with 6-0 Vicryl (Figure 5). Histologic examination of the specimen showed stratified squamous epithelium. At 9-month follow-up, the patient was symptom-free without intervention therapy. Informed consent was obtained before from her parents for this report.

## 3. Discussion

Although bilateral CLFs have been reported in the literature [2, 4, 7], they are often unilateral and located inferonasal to the medial canthal angle, as it was in our case. Patients with CLF usually present with epiphora or discharge. The nasolacrimal system is usually patent [1]. The clinical presentation may delay for many years after birth due to the evaporation of small amounts of discharge [8]. The two main complications are chronic local eczema due to maceration and chronic or acute dacryocystitis due to ascending infection [5]. Our case had discharge from the orifice of fistula especially in windy days and had no associated infectious complication.

The embryogenesis of the nasolacrimal system begins as a stiffening of ectoderm in the nasooptic fissure that invaginates between the maxillary and frontonasal processes, giving rise to the canaliculi proximally and the lacrimal sac and nasolacrimal duct distally. Canalization of the buried ectodermal cord occurs throughout the length of the nasolacrimal apparatus. Incomplete separation of the cord from the surface epithelium or abnormal out-budding of the buried ectodermal cord can result in supernumerary canaliculi [9].

CLFs have been systemically described with ectrodactyly-ectodermal dysplasia-clefting syndrome [10], hypospadias [11], Down syndrome [12–15], uterus didelphys [16], and VACTERL (vertebral anomalies, anal atresia, cardiac defects, tracheoesophageal fistula and/or esophageal atresia, renal and radial anomalies, and limb defects) [17] in some previous reports [1, 18]. There are two cases that were reported to be associated with renal agenesis in the literature. Beside renal agenesis, one of them had also multiple anomalies [17] and the other one had uterus didelphys [16]. In addition to right renal agenesis, our case had also left ectopic pelvic kidney that makes it unique in the literature because, according to our knowledge, this is the first reported case of CLF accompanied with ectopic pelvic kidney. Our case had no cardiac, vertebral, esophageal, or limb anomaly. CLFs have been also reported with some ocular associations such as stenosis in the lacrimal tract, strabismus, and hypertelorism [11, 18, 19]. Our case had no further ocular abnormality.

The choice of treatment for CLF is controversial. A wide range of treatment options for symptomatic CLF have been discussed in the literature, like cauterization, excision of the fistula, and excision with intubation alone or in combination with dacryocystorhinostomy [20–23]. We managed our case successfully by primary fistulectomy without dacryocystorhinostomy.

In conclusion, CLF is a rare anomaly that might be associated with important systemic life threatening conditions. That is why the patients with CLF should be evaluated carefully for any possible associated anomalies like renal agenesis and ectopic kidney.
